# Evaluation of Drug Labels Following the 2015 Pregnancy and Lactation Labeling Rule

**DOI:** 10.1001/jamanetworkopen.2020.15094

**Published:** 2020-08-31

**Authors:** John J. Byrne, Alexander M. Saucedo, Catherine Y. Spong

**Affiliations:** 1 Division of Maternal-Fetal Medicine, Department of Obstetrics and Gynecology, University of Texas Southwestern Medical Center at Dallas

## Abstract

**Importance:**

The US Food and Drug Administration (FDA) Pregnancy and Lactation Labeling Rule (PLLR), implemented in 2015, includes information on pregnancy, lactation, and women and men with reproductive potential.

**Objectives:**

To identify the drugs that have adhered to the new PLLR format; to shed light on the continued need for implementation of pregnancy, lactation, and reproduction into clinical studies; and to evaluate how many new therapeutic products have human and animal data specific to pregnancy and lactation.

**Design, Setting, and Participants:**

This cross-sectional study of 290 new therapeutic drugs reviewed labeling data for newly FDA-approved therapeutic products from January 2010 to December 2019. Therapeutic products submitted on or after June 30, 2015, were required to be in PLLR format; those approved from June 30, 2007, to June 29, 2015, had until June 30, 2019, to be in PLLR format. Approval data and subsequent labeling revision were evaluated for pregnancy and lactation data (human and animal), pregnancy registry, black-box warnings, and inclusion of PLLR labeling format.

**Exposures:**

Date of new drug approval by FDA.

**Main Outcomes and Measures:**

Compliance with PLLR; presence of animal or human data; presence of pregnancy registries; and presence of information regarding female and male reproductive potential.

**Results:**

A total of 290 new molecular entities or therapeutic products were approved by the FDA between 2010 and 2019 in 19 categories. Black-box warnings occurred in 89 drugs (30.7%; 95% CI, 25.4%-36.3%), with 3 (3.4%; 95% CI, 0.7%-9.5%) involving pregnancy. All products submitted after June 30, 2015, were in PLLR format; however, of the 138 submitted between 2010 and that date, 45 (32.6%; 95% CI, 24.9%-41.1%) were not in PLLR format by June 30, 2019. During the 10 years of data analyzed, significantly more were in PLLR format (*P *for trend < .001). Most approved therapeutic products have pregnancy data derived from animal studies (260 products; 89.7%; 95% CI, 85.6%-92.9%) but only 31 (10.7%; 95% CI, 7.4%-14.8%) derived data from human studies. Only 148 therapeutic products (51.0%; 95% CI, 45.1%-56.9%) had any data associated with lactation, 143 (49.3%; 95% CI, 43.4%-55.2%) originating from animal studies and 8 (2.8%; 95% CI, 1.2%-5.4%) from human studies.

**Conclusions and Relevance:**

The results of this study show that with the implementation of PLLR in the last decade, new therapeutic products were in compliance with the new rules; however, more than one-third of labels remain out of PLLR compliance. Human data on pregnancy and lactation are available in less than 20% of new product labeling.

## Key Points

**Question:**

What is the current state of US Food and Drug Administration labeling of medications in relation to pregnancy and lactation?

**Findings:**

In this cross-sectional study of 290 newly approved medications from January 2010 to December 2019, all products submitted after June 20, 2015, were in compliance with the Pregnancy and Lactation Labeling Rule (PLLR); however, of those submitted between 2010 and 2015, 32.6% were not in PLLR format by the designated date of June 30, 2019. Human data on pregnancy and lactation were available in less than 20% of new product labeling.

**Meaning:**

This study found that with the implementation of PLLR in the last decade, new therapeutic products are in compliance with the new rule; however, more than one-third of labels remain out of PLLR compliance.

## Teaser

This cross-sectional study identifies the drugs that have adhered to the new US Food and Drug Administration Pregnancy and Lactation Labeling Rule, sheds light on the continued need for implementation of pregnancy, lactation, and reproduction into clinical studies, and evaluates how many new therapeutic products have human and animal data specific to pregnancy and lactation.

## Introduction

Most women will take at least 1 medication during pregnancy, delivery, or the postpartum period.^1,2^ To inform patients on the safety and efficacy of these therapies used during pregnancy and postpartum, obstetrical care professionals rely on available information. The criterion standard for this information is the US Food and Drug Administration (FDA) drug label. The FDA evaluates new and existing medications and provides recommendations regarding safety, specifically during pregnancy and lactation. These recommendations were initially provided in the late 1970s, with the development of the alphabetic risk stratification system (ie, A, B, C, D or X, with A indicating adequate and well-controlled studies have proven safety in pregnancy and X indicating that studies have shown human fetal risk if ingested during pregnancy).^3,4^ This approach was designed to convey the type and amount of data that were available at the time. However, this design led clinicians to misinterpret the recommendations as a grading system of risk and not a categorization of available data. Nearly 2 decades later in 1994, the FDA established the Pregnancy Labeling Task Force in an attempt to provide a more thorough risk assessment of medications’ effects on pregnancy.^5^ This attempt to risk stratify medications for pregnancy was further heightened with the establishment of pregnancy registries in 2002 to promote clinical trials examining use of new medications by pregnant women.^6^ The introduction of a new FDA labeling requirement was posted in the Federal Register in 2008, which would eventually become the implementation of the Pregnancy and Lactation Labeling Rule (PLLR) in 2015.^4,5^ The change in the labeling process transitioned from the initial letter grade pregnancy category to a more structured approach. The goal was to aid prescribers and patients in obtaining a greater understanding of the nuances of the available data that exist. The new PPLR format includes available data summaries as well as the strength of these data. Furthermore, the data provided include information on pregnancy, lactation, and exposure registries, along with a new subsection for women and men with reproductive potential. The subsection on women and men with reproductive potential includes information regarding pregnancy testing, contraception, and infertility as it relates to the drugs, when necessary.^4^

The implementation schedule of the new PLLR categorization required that FDA drug submissions on or after June 30, 2015, be in the new PLLR format. The drugs that were approved between June 30, 2007, and June 29, 2015, were to have transitioned to the new PLLR format by June 30, 2019.^7^ Additionally, the applications approved before June 30, 2001, that were in the previous labeling format were not required to transition to the PLLR format. However, they were required to remove the pregnancy letter category by June 29, 2018. The consequences of the PLLR initiative has yet to be determined. Specifically, it is not known how many new and preexisting FDA drug labels have been transitioned to the PLLR format nor the quality of the data that exist for pregnancy, lactation, and reproductive potential. We have 3 aims in this study, as follows: (1) to identify the drugs that have adhered to the new PLLR format; (2) to shed light on the continued need for implementation of pregnancy, lactation, and reproduction in clinical studies; and (3) to evaluate how many new therapeutic products have human and animal data specific to pregnancy and lactation. We hypothesized that all new products submitted after June 30, 2015, would be in compliance with the PLLR format and that less than half of new products would have human data specific to pregnancy and lactation.

## Methods

This cross-sectional study evaluates labeling data available for all new, original drug approvals between January 1, 2010, and December 31, 2019. An online, publicly available database maintained by the FDA houses all initial labeling information and any subsequent labeling updates.^8^ The database was queried for all drug approvals and labeling updates. The labeling information for all new drugs was extracted from the database. New formulations, generic approvals, supplemental applications, abbreviated new drug approvals, and new biologicals were excluded from the analysis. Our study was reviewed by the institutional review board of the University of Texas Southwestern Medical Center and Parkland Hospital and was considered exempt given that there was no more than minimal risk. This study followed the Strengthening the Reporting of Observational Studies in Epidemiology (STROBE) reporting guideline.

The initial label and subsequent labeling revisions of new molecular entities (classified as type 1 by FDA) were identified and reviewed on August 1, 2019. This process was completed by 2 separate investigators (J.J.B and A.M.S.) to minimize bias. The data extracted included time of original approval (month and year), drug class, therapeutic category, quality of breastfeeding data, quality of pregnancy data, data on female and male reproductive potential, pregnancy and lactation registries, black-box warnings, and inclusion of the PLLR labeling format. If the medication was not yet transitioned to the PLLR labeling format, the pregnancy class was recorded. Potential markers of fetal toxic effects were recorded, which included black-box warnings for fetal toxic effects and teratogenicity. The subsequent labeling revisions were reviewed for updates on March 18, 2020, by 1 of us (J.J.B.). Any changes from the initial update were also noted, and the date of the labeling update was recorded.

The sources of the breastfeeding data and pregnancy data were recorded. This included origin of data (ie, human data, animal data, or extrapolation from other sources, including similar mechanism of action). The presence of pregnancy registries was evaluated as well as whether this information was specific to the agent or a general drug registry for specific syndromes. Additionally, the presence of fast-track drug approval status was evaluated.^9^

### Statistical Analysis

Statistical analysis was performed using χ^2^ and Mantel-Haenszel test for trend as appropriate. Analysis was conducted in SAS statistical software version 9.4 (SAS Institute), with a 2-tailed *P* < .05 considered significant.

## Results

There were 290 new molecular entities and therapeutic products approved between 2010 and 2019 in 19 categories (Table 1). There were 138 new drug approvals (47.6%) from January 1, 2010, to June 30, 2015, and 152 products (52.4%) approved from July 1, 2015, to December 31, 2019. The largest of these therapeutic categories were oncology, with 70 new drugs (24.1%), and infectious diseases, with 55 new drugs (19.0%). Black-box warnings were identified in 89 drugs (30.7%) (Table 1). The therapeutic categories with the greatest number of black-box warnings were oncology (15 [16.9%]) and infectious diseases (17 [19.1%]). Black-box warnings related to pregnancy were noted in 3 newly approved products (3.4%; 95% CI, 0.7%-9.5%) (Table 1).

**Table 1.** Therapeutic Categories, Approvals, Black-Box Warnings, and Reproductive Black-Box Warnings of New Drug Approvals

Therapeutic category	Frequency (%)		
	Approvals	Warning	
		Black box	Reproductive black box
Anesthesia and analgesia	1 (0.3)	0	0
Cardiology	7 (2.4)	1 (1.1)	1 (11.1)
Dermatology	4 (1.4)	0	0
Diagnostic imaging	10 (3.4)	3 (3.4)	0
Endocrine	12 (4.1)	5 (5.6)	0
Gastrointestinal	18 (6.2)	5 (5.6)	0
Genetics	5 (1.7)	0	0
Hematology	17 (5.9)	10 (11.2)	1 (11.1)
Immunology	2 (0.7)	0	0
Infectious disease	55 (19)	17 (19.1)	1 (11.1)
Nephrology	7 (2.4)	4 (4.5)	0
Neurology	36 (12.4)	7 (7.9)	1 (11.1)
Obstetrics and gynecology	8 (2.8)	4 (4.5)	0
Oncology or radiology oncology	70 (24.1)	15 (16.9)	3 (33.3)
Ophthalmology	5 (1.7)	0	0
Psychiatry	11 (3.8)	10 (11.2)	0
Pulmonary	16 (5.5)	5 (5.6)	2 (22.3)
Rheumatology	4 (1.4)	3 (3.4)	0
Urology	2 (0.7)	0	0
Total	290 (100)	89 (30.7)	9 (3.1)

**Figure 1.**
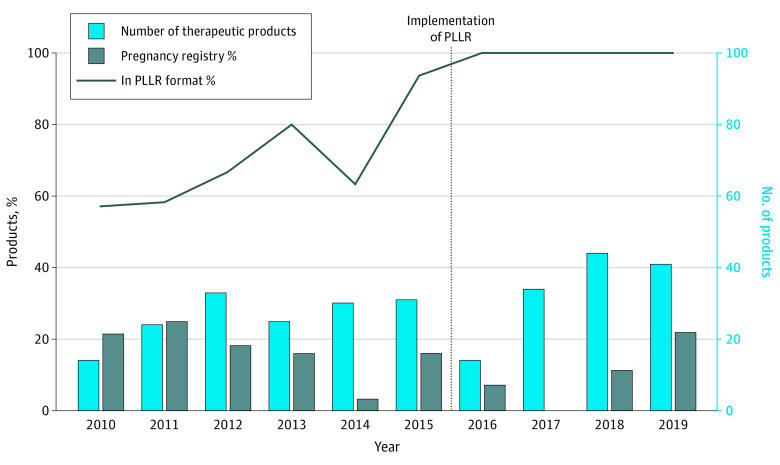
Association of the Pregnancy and Lactation Labeling Rule (PLLR) With Drug Labeling The number of new products approved by the FDA ranged from 14 (2010, 2016) to 44 (2018). All products submitted after June 30, 2015, were in PLLR format; however, of those submitted between 2010 to 2015, 45 of 138 (32.6%) were not in PLLR format by the designated date of June 30, 2019. The percentage that included a pregnancy registry ranged from 0% to 25%.

Notably, all new molecular entities and therapeutic products submitted after June 30, 2015, were in the PLLR format (Figure 1). However, 45 of 138 approved submissions (32.6%; 95% CI, 24.9%-41.1%) before June 30, 2015, were not in PLLR format by June 30, 2019 (Figure 1). By January 1, 2020, this number decreased to 26 (18.8%). Overall, 15.5% of all new molecular entities during our study period were not in PLLR format by the deadline of June 30, 2019. During the 10 years, significantly more submissions were in the PLLR format (*P *for trend < .001). Of the 92 medications from 2012 to 2019 that were newly approved through the fast track designated status, only 7 (7.6%) were not in compliance by June 30, 2019.

**Figure 2.**
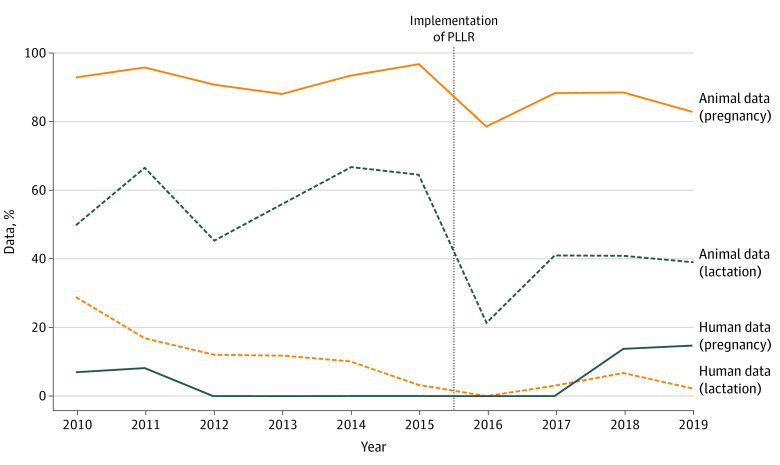
Pregnancy and Lactation Data Derived From Human and Animal Studies Before and After Implementation Date The amount of human and animal data for pregnancy and breastfeeding ranged from year to year. The amount of pregnancy and lactation-specific data derived from human research ranged from 0% to 28% and 0% to 8%, respectively. The amount of pregnancy and lactation-specific data derived from animal research ranged from 79% to 100% and 39% to 68%, respectively. The Pregnancy and Lactation Labeling Rule (PLLR) was implemented June 30, 2015.

Most therapeutic products that were approved had associated data regarding the use of medication in pregnancy. Although only 31 approved therapeutic products (10.7%; 95% CI 7.4%-14.8%) were found to have human data related to pregnancy, 260 (89.7%; 95% CI, 85.6%-92.9%) were found to have animal data associated with pregnancy (Figure 2). The drug categories with the highest amount of human data related to pregnancy included genetics (4 of 5 [80.0%]) and obstetrics and gynecology (3 of 8 [37.5%]). The drug categories with the highest amount of animal data related to pregnancy included anesthesia and analgesia, dermatology, genetics, hematology, immunology, infectious diseases, psychiatry, rheumatology, and urology (all 100%).

Similarly, when examining data related to lactation, 141 of 290 therapeutic agents (48.6%) approved had no data regarding medication safety. Only 8 products (2.8%; 95% CI, 1.2%-5.4%) were found to have any associated human data, and 143 (49.3%; 95% CI, 43.4%-55.2%) were found to have associated animal data for lactation. The drug categories with the highest number of human data related to lactation were obstetrics and gynecology (2 of 8 [25.0%]) and psychiatry (1 of 11 [9.1%]). The drug categories with the highest amount of animal data related to lactation were anesthesia and analgesia (1 of 1 [100.0%]) and rheumatology (4 of 4 [100.0%]), endocrine (10 of 12 [83.3%]), and pulmonology (14 of 16 [87.5%]). There has not been a substantial increase in pregnancy data arising from human or animal research after the transition to the PLLR rule. There was no difference in the amount of data arising from human studies in lactation between pre-PLLR and post-PLLR periods; however, the amount of data derived from animal studies decreased after institution of the PLLR rule (*P *for trend = .01).

**Figure 3.**
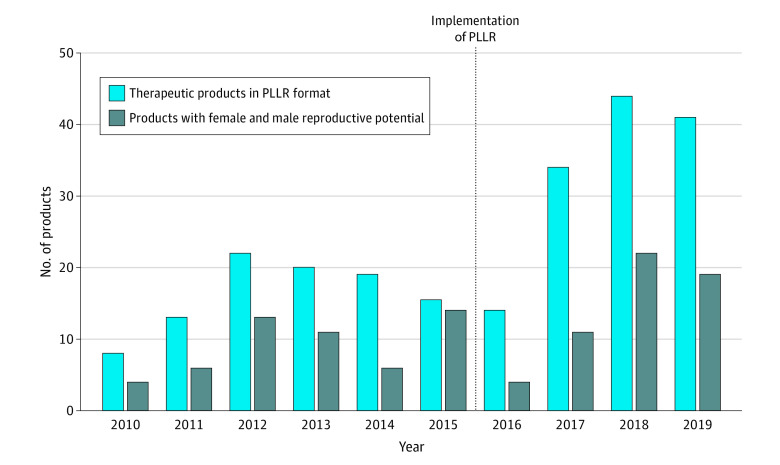
Products With Female and Male Reproductive Potential Category Products with data in the female and male reproductive potential subsection by year as compared with therapeutic products in Pregnancy and Lactation Labeling Rule (PLLR) format. The PLLR was implemented June 30, 2015, and is highlighted on the figure.

The PLLR final rule includes a subsection 8.3, female and male reproductive potential, to include information for populations when recommendations for pregnancy testing or contraception related to the therapy are needed or there are data suggesting the therapy affects fertility or preimplantation loss. This subsection was incorporated in 115 of 290 labels (39.7%; 95% CI, 34.0%-45.5%) of all approved therapeutic products (Figure 3), and 110 of 245 (44.9%) of those that transitioned to the PLLR format by June 30, 2019. Lastly, pregnancy registries were noted in 40 newly approved therapeutic products (13.8%) (Table 2).

**Table 2.** Pregnancy Registries Seen in New Drug Approvals by Therapeutic Category

Therapeutic category	Pregnancy registries, No. (%) (N = 40)
Anesthesia or analgesia	0
Cardiology	0
Dermatology	0
Diagnostic imaging	0
Endocrine	0
Gastrointestinal	2 (5.0)
Genetics	1 (2.5)
Hematology	1 (2.5)
Immunology	0
Infectious disease	10 (25.0)
Nephrology	0
Neurology	14 (35.0)
Obstetrics and Gynecology	2 (5.0)
Oncology or radiology oncology	1 (2.5)
Ophthalmology	0
Psychiatry	7 (17.5)
Pulmonary	0
Rheumatology	2 (5.0)
Urology	0

## Discussion

There were 2 primary observations from the analysis. First, while there is 100% compliance with the PLLR update since the implementation date of June 30, 2015, not every FDA label has been converted. More than one-third of older submissions still need to be transitioned to this new format. Second, our study demonstrated the limited amount of data associated with pregnancy and lactation, with most available data originating from animal studies.

The implementation of PLLR has resulted in new molecular entities and therapeutic products in compliance with the new format; however, this is a relatively new development. Mazer-Amirshahi and colleagues^10^ evaluated changes in labeling for new molecular entities and selected biological agents approved from 2003 to 2012, prior to the implementation of PLLR in 2015. They identified only 10 medications that were incorporated into the new labeling format. The differences noted in this current study are reflective of the updated period and the exclusion of biological agents. Barrow^11^ evaluated details of embryo-fetal development for all FDA drug labels approved in 2016 and 2017 and noted that only 2 drugs were initially not placed in PLLR format. However, 1 was subsequently converted per the author. Our study has confirmed that all drug labels after the June 30, 2015, deadline have been transitioned to the new format. Unfortunately, not all drug labels before that time have been converted, therefore limiting the initial intent of the conversion.

The lack of information regarding medication use in pregnancy and lactation has been highlighted as an area of need in prior studies.^10,12,13,14^ Mazer-Amirshahi et al^10^ evaluated trends in FDA labels specific to pregnancy from January 2003 to December 2012. Although an 8-year difference in study period exists between our study and theirs, there was no change in the amount of data associated with lactation, with 48% of drugs with no associated data in their cohort and ours. Most lactation-specific data in both studies were found to arise from research on animals. Interestingly, there were more lactation-specific data derived from animal studies prior to PLLR; however, it is unlikely to be caused by the implementation of PLLR. Lastly, animal lactation data typically do not reliably estimate levels in human milk because of species-specific differences in lactation physiology.^15^

Similar to lactation-associated data, Mazer-Amirshahi and colleagues^10^ showed that most approved drugs had pregnancy-associated data arising from animal studies (93% in Mazer-Amirshahi et al vs 90% in our study) but few with data arising from human studies. It must be mentioned that the FDA actively encourages the inclusion of pregnant women in clinical trials.^16^ Unfortunately, these findings reveal that the trend of the few clinical trials involving pregnant women continues.

### Limitations and Strengths

This study should be interpreted in the context of its limitations. This is a cross-sectional study; therefore, it only evaluates the data at 2 distinct points. The FDA maintains this database, and although most drugs are updated frequently, there can be a lag time between new data generation and the creation of a label update. In addition, several of the drugs were approved for conditions that only pertain to men (ie, prostate cancer). The strength of the study is that it evaluated a large database of newly approved prescription drugs, which require screening by the FDA before becoming available in the United States. Additionally, this study evaluated the transition of medications to the PLLR format before and after the deadline, which has not to our knowledge been performed previously.

## Conclusions

The findings of this study show that with the evolution of the PLLR format, great strides have been made in providing strong evidence-based recommendations and information for practitioners and patients. The effects of this PLLR format over time will be interesting to follow to determine whether new registries, human data, and clinical trials are created to provide information for this population. These changes will need to continue to be promoted by the pharmaceutical industry, health care professionals, insurance agencies, and patients. The critical dearth of human data specific to pregnancy and lactation on new therapeutic products cannot be understated.
